# Camera Configuration for Wearable Electronic Travel Aids Supporting Urban Mobility of Visually Impaired People

**DOI:** 10.3390/s26185841

**Published:** 2026-09-15

**Authors:** Yancheng Li, Jiajun Zheng, Jinjing Zhao, Xuantuo Huang, Yiding Liu, Caiyi Wang, Jingjing Xu, Shengyong Xu

**Affiliations:** 1 Key Laboratory for the Physics and Chemistry of Nanodevices, School of Electronics, Peking University, Beijing 100871, China; lycheng@pku.edu.cn (Y.L.); 2401112206@stu.pku.edu.cn (J.Z.); zhaojinjing@pku.edu.cn (J.Z.); 2301213327@pku.edu.cn (Y.L.); 2 School of Integrated Circuits, Shandong University, Jinan 250010, China; 202332369@mail.sdu.edu.cn

**Keywords:** visual impairment, electronic travel aids, wearable cameras, camera configuration, field of view, sensing coverage, hazard visibility, obstacle avoidance

## Abstract

Visually impaired people face urban mobility hazards such as vehicles, pedestrians, curbs, and steps. Wearable electronic travel aids can support obstacle avoidance only when these hazards fall within sensing coverage. Existing studies have focused mainly on algorithms and human–computer interaction, while the sensing coverage required by real-world hazards remains less examined. We collected 1486 min of street-scene video, built urban mobility hazard samples, and used simulation to compare target pixel counts over time for cameras with different horizontal fields of view. Among 81,353 valid samples, hazards showed concentrated high-frequency categories and a rich long tail, mainly including vehicles, scooters, pedestrians, curbs, bollards, poles, trees, and steps. Simulation showed that a horizontal field of view of approximately 120° balanced pixel coverage and sustained observation. We then built a chest-mounted four-camera prototype and conducted a feasibility demonstration at a real intersection. Camera configuration should therefore be guided by real-world hazard visibility, and coordinated multi-camera coverage is a promising design choice for wearable ETAs in urban environments.

## 1. Introduction

Visually impaired people (VIPs) face many barriers in daily life, especially when traveling outside the home. In 2020, approximately 596 million people worldwide lived with vision impairment [1]. Yet, VIPs appear far less often in everyday urban travel than this number would suggest, even in cities with basic accessibility infrastructure such as tactile paving. For nearly a century, researchers have explored technologies that support independent mobility. Recent reviews have summarized progress in assistive navigation systems and related travel aids [2,3,4,5,6,7]. Products and services such as Be My Eyes, TapTapSee, Aira, and DotLumen have also entered practical use. Even so, independent mobility remains a global challenge for this population, and addressing it would substantially improve their quality of life.

The main difficulty lies in the complexity of the urban traffic environments in which VIPs travel. Even in a simple static obstacle-avoidance scenario, VIPs may encounter curbs or other tripping hazards as soon as they deviate from tactile paving. They also need to cross roads with substantial vehicle and pedestrian traffic. In these situations, they must pass through areas with few tactile ground cues and within a limited time [8]. The challenge is even greater at unsignalized intersections with heavy traffic.

**Figure 1:**
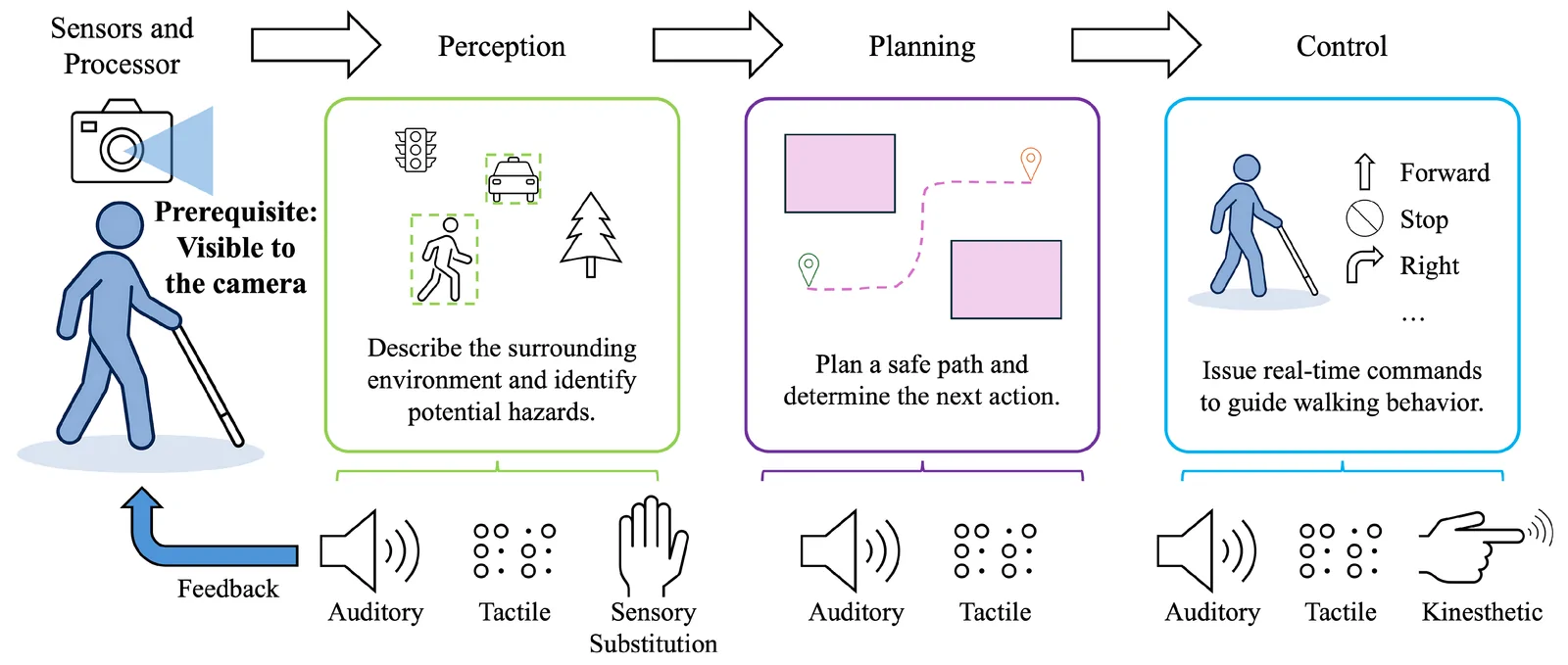
Classification of electronic travel aids based on their output to users. Within the perception, planning, and control pipeline, these systems provide environmental descriptions, route decisions, or real-time action instructions. Regardless of whether the feedback is delivered through speech, haptic feedback, sensory substitution, or kinesthetic feedback, the front-end sensors must first capture mobility hazards within the camera view before the system can help users avoid obstacles.

Most proposed solutions for VIP mobility rely on electronic systems that integrate technologies such as artificial intelligence, wireless communication, large-scale data processing, and human–computer interaction. Such systems are known as electronic travel aids (ETAs) [9]. By analogy with the perception–planning–control pipeline in autonomous driving, these systems can be grouped by their mode of assistance into the three categories shown in Figure 1 [10]. The first category provides environmental information through speech or sensory substitution, helping users perceive their surroundings [11,12]. The second category performs path planning based on perceived environmental information and conveys the walking route through speech, haptic feedback, or related modalities [13,14]. The third category goes further by directly instructing users how to move through speech, haptic feedback, or kinesthetic feedback, effectively serving a control function [15,16]. A single device may combine more than one of these functions. For example, a device operated by a remote sighted agent (RSA) can use the human agent’s speech to describe the environment, explain the route, and issue immediate action instructions [17,18].

However, all three categories can help VIPs navigate urban environments only if the front-end sensors first capture mobility hazards within their sensing range [8,10]. Existing work has focused mainly on downstream modules, including localization, perception, planning, and control. Much of this work emphasizes sensor selection, multisensor fusion, and planning and control algorithms to improve system accuracy, robustness, and real-time performance [5]. The visibility of mobility hazards is usually left implicit in individual system designs and has rarely been treated as a central research question.

Among sensing modalities for wearable devices, visual sensors, namely cameras, are a natural focus for studying sensing coverage because they are easy to mount, low-cost, and information-rich [19]. For wearable travel aids, however, camera configuration should account not only for sensing coverage but also for human factors and wearability because sensor placement and device form affect comfort, stability during movement, usability, and compatibility with everyday mobility aids [10,20]. Human-centred visual-assistance research likewise emphasizes the need to align wearable hardware and interaction design with user needs [21]. Camera configuration should therefore be treated as an early system-design decision that connects sensing coverage with the practical requirements of wearable deployment.

Although camera configuration and placement have been studied for ETAs, existing work has not analyzed them in relation to the real hazards that VIPs face during urban travel. Previous studies have examined individual aspects of camera configuration in specific assistive tasks. Manduchi and Coughlan [22] found that, during last-meter guidance with a handheld camera, increasing the FOV facilitated target acquisition but reduced target resolution. Iwamura et al. [23] found that visually impaired users preferred a wide-FOV or omnidirectional camera for object search, while Son and Weiland [8] reported that the limited FOV of an earlier head-mounted crosswalk system made it difficult to keep the traffic signal in view. Varshney et al. [24] examined how sensor vantage affects outdoor navigation algorithms for ETAs, but did not derive the required sensing coverage from urban mobility hazards. Related fields have also explored optimal camera configuration [25,26]. Overall, these studies have not directly addressed the urban mobility needs of VIPs.

In this study, we investigate camera configuration for ETAs that support VIP mobility in urban environments. We first collected a large set of street-scene video data and systematically analyzed the mobility hazards that VIPs may encounter. We then built a simulation environment with a 3D game engine to reproduce urban travel scenarios and examine how different camera configurations affect sensing coverage. Finally, guided by the simulation results, we built a four-camera prototype that covers the forward, downward, and lateral views. We demonstrated its use in helping VIPs cross a busy road under remote sighted assistance.

The remainder of this paper is organized as follows: Section 2 describes the methods. Section 3 presents the results of the urban mobility hazard analysis, the camera sensing coverage experiments in simulation, and the four-camera prototype demonstration for road crossing. Section 4 discusses the findings and their implications. Section 5 concludes this paper.

## 2. Methods

This study followed the following workflow: First, we used real-world video recordings to systematically analyze mobility hazards in urban street scenes. Specifically, we used a vision-language model (VLM) to identify elements in the recorded images that could affect VIP mobility, and manually reviewed sampled outputs to compile the hazard list. Next, we built a simulation environment in a 3D game engine and measured the pixel-level characteristics of hazards captured by cameras with different horizontal field of view (FOV) settings. Finally, guided by the simulation results, we developed a chest-mounted four-camera prototype for remote sighted assistance and demonstrated its use for helping VIPs cross a road in a real street environment.

### 2.1. Urban Mobility Hazard Analysis

**Figure 2:**
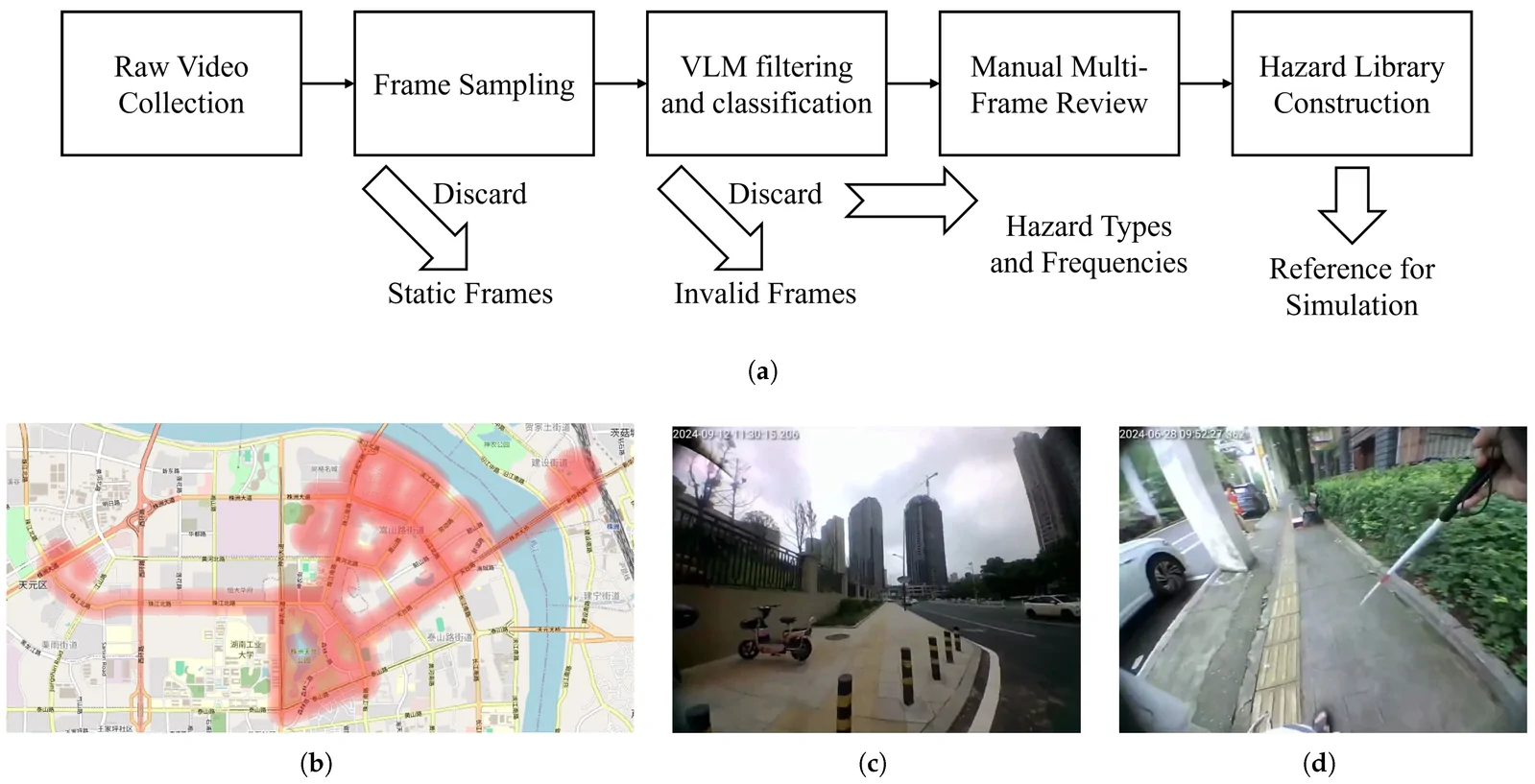
Workflow for constructing the urban video dataset and generating the hazard library. (**a**) Processing pipeline from raw video collection, frame sampling, and VLM-based screening and classification to manual multi-frame review and hazard library construction. (**b**) Areas in Zhuzhou, Hunan Province, China where video data were collected. Red indicates the areas covered by data collection. Chinese labels on the map denote geographic place names provided by the map service and are not study-specific annotations. (**c**,**d**) Representative street-scene frames captured by the chest-mounted recording device.

To analyze the mobility problems that VIPs may encounter as realistically and comprehensively as possible, we collected video while walking through urban streets with a simple chest-mounted recording device. Data collectors worked within the street network of Zhuzhou, Hunan Province, China (Figure 2b). Each recording started from a fixed location, followed the sidewalk along a selected street, and returned to the same location. Example frames from the collected videos are shown in Figure 2c,d. The image quality was sufficient only for humans and computer vision algorithms to identify objects in the scenes, and was far below surveying and mapping standards.

The urban scenes captured in these videos provided a solid basis for analyzing the mobility hazards that VIPs may encounter. To identify these hazards as objectively as possible, we used a VLM as a research aid. The workflow is described below and shown in Figure 2a.

We sampled frames from the raw videos. For both humans and computer vision algorithms, reliable object recognition in contextual images generally requires an object to occupy at least 32×32 pixels [27,28]. Reduced to one dimension, this means that objects whose feature length reaches this threshold should not be missed. A reasonable sampling interval can therefore be estimated from the distance at which the smallest object just occupies the minimum required number of pixels. Assume that the length of an object along the image width, used as the feature length, is $l_{\mathrm{obj}}$, the image width is *p* pixels, and the desired object width in the image is $p_{\mathrm{obj}}$ pixels. Also assume that the horizontal FOV is θ and that the object is centered in the camera view. From triangle geometry, the distance from the camera optical center to the object when it occupies $p_{\mathrm{obj}}$ pixels along the image width is denoted as $d_{\mathrm{obj}}$ and can be calculated using Equation (1).

(1) $$d_{\mathrm{obj}}=\frac{l_{\mathrm{obj}}}{2}\cdot \frac{p}{p_{\mathrm{obj}}}\cdot \frac{1}{\tan\frac{\theta }{2}}$$

Using $l_{\mathrm{obj}}=0.3\;m$, p=640pixels, $p_{\mathrm{obj}}=32\;\mathrm{pixels}$, and θ=135°, Equation (1) gives $d_{\mathrm{obj}}=1.2\;m$. To account for coverage gaps in the camera view and possible missed objects between sampled frames, we doubled the sampling rate implied by this result. Assuming a pedestrian speed of approximately 1m/s, the current frame was sampled when more than 0.6s had elapsed since the previous sampled frame. To exclude periods when the camera was stationary, sampling was skipped if the mean absolute pixel difference between the current frame and the previous sampled frame showed no clear change. Each sampled frame was then used as an image input to the VLM (Qwen 3 VL 30B, 4-bit quantized), together with a text prompt. The expected output was a JSON text that listed the outdoor mobility hazards in the image by category and by short natural-language phrase. The categories were static_obstruction, moving_obstruction, overhang_obstruction, and step_or_dropoff. These categories were defined mainly by obstacle height. Ground-level dynamic obstacles, such as pedestrians and moving vehicles, were treated as a separate category.

To ensure that the hazards evaluated in simulation were representative, we first calculated the occurrence frequency of outdoor mobility hazard categories. We then used multiple-frame sampling to select 100 images from frames containing these hazards. The specific hazards in these images were manually recorded and used to form the hazard list. In the urban simulation environment, we selected common hazard occurrences from this list for analysis. This procedure helped align the simulated hazards with those encountered by VIPs during urban travel.

### 2.2. Visibility Simulation of Camera Configurations

To analyze how a key camera-configuration parameter affects hazard visibility under controlled conditions, we used an urban scene from the Downtown West Modular Pack in Unreal Engine as the simulation environment. In the first experiment, we selected a fixed route that contained typical elements of urban travel. For each high-frequency hazard category in the hazard list, we selected one representative instance as the analysis target. The second experiment simulated a vehicle approaching laterally and compared a single forward-facing camera with a three-camera configuration that covered the forward, left, and right views. The third experiment used a route containing a fire hydrant and a low bollard to compare a single forward-facing camera with a two-camera configuration that covered the forward view and a view directed 60° downward.

Using the API provided by AirSim [29], we set the initial camera position, motion speed, and sampling duration before each run. Within each experiment, the motion trajectories and sampling duration were identical across camera configurations. In the first and third experiments, the camera moved along the prescribed route at a constant speed. In the second experiment, the camera remained stationary while the simulated vehicle approached from the side. The first experiment evaluated the single-camera configuration at horizontal FOVs from 90° to 170° in 10° increments. The second and third experiments evaluated the multi-camera configurations at horizontal FOVs from 90° to 140° in 10° increments. The narrower FOV range used in the second and third experiments was selected based on the results of the first experiment. This design allowed us to examine how horizontal FOV and coverage in different viewing directions affected hazard visibility.

In the simulation, all camera distortion parameters were set to zero, and imaging followed an ideal pinhole model. In practice, cameras with wide FOVs usually use fisheye lenses. Their projection model differs from that of a pinhole camera, and they often introduce substantial radial distortion. However, the primary metric at this stage was visibility. For an imaging system with fixed resolution, the pixel count of a target is mainly determined by its angular size and the camera’s angular sampling rate. If the simulated and real cameras have similar angular sampling rates in the region of interest under comparable resolution and FOV settings, the simulated target pixel count can still serve as an approximate reference. Such pixel counts can approximate visibility and recognizability for real cameras. At the same time, pinhole projection under an ultra-wide FOV produces clear perspective exaggeration, nonuniform scaling near the image edges, and shape stretching. These effects can partly reflect the visual appearance and recognition difficulty introduced by real wide-angle imaging.

### 2.3. Chest-Mounted Four-Camera Prototype and Field Demonstration

**Figure 3:**
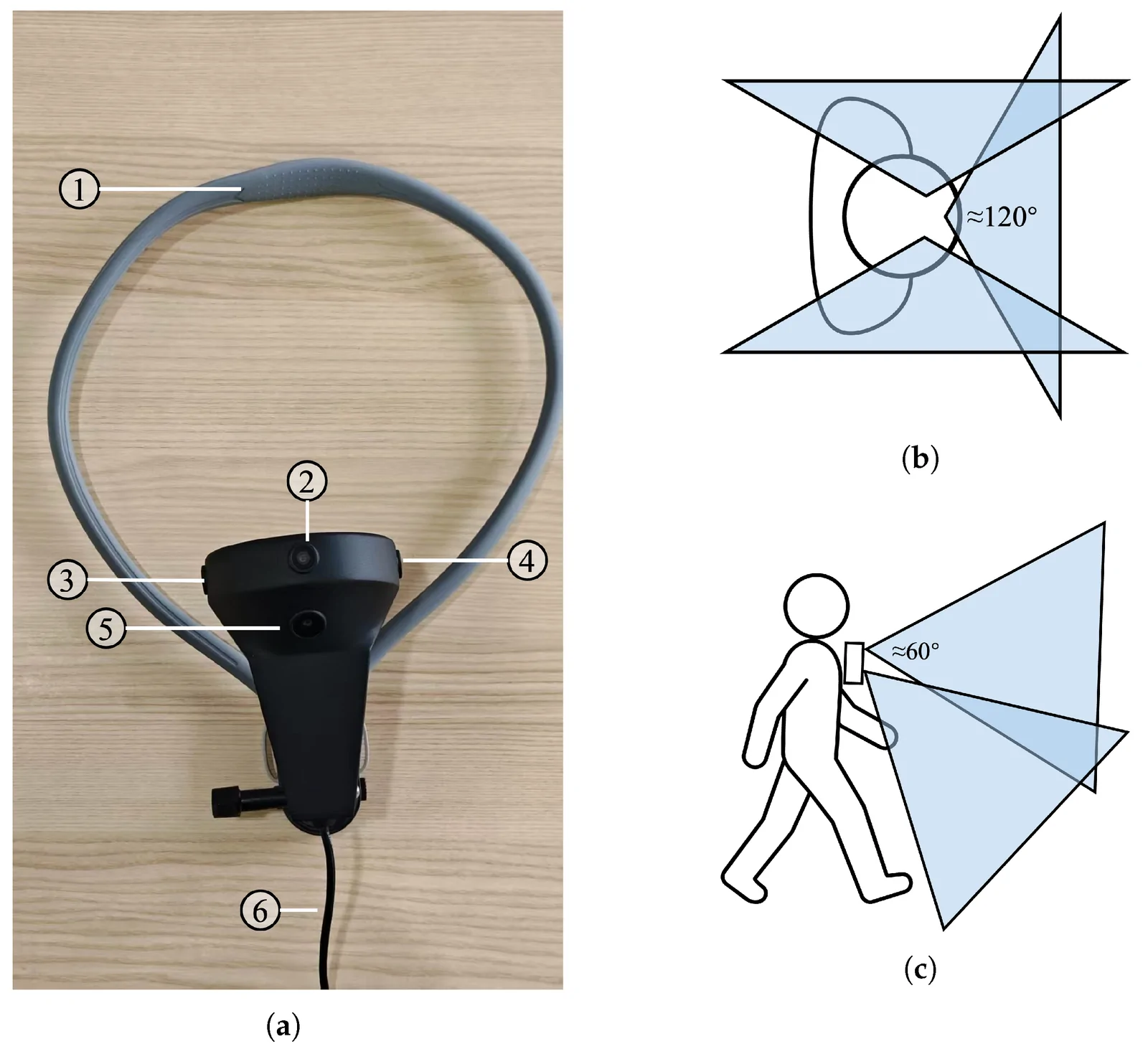
Chest-mounted four-camera prototype and its effective FOV. (**a**) Photograph of the prototype. Labels 1–6 indicate the neck-hanging structure, forward-facing camera, right-facing camera, left-facing camera, downward-facing camera, and USB cable, respectively. (**b**) Top view. Each camera provides an effective horizontal coverage of approximately 120°. The effective horizontal coverage of the left and right cameras is reduced by occlusion from the head and shoulders. (**c**) Side view. Each camera provides an effective vertical coverage of approximately 60°.

Based on the trade-off between FOV and visibility observed in the simulation, the prototype did not use a single ultra-wide-angle camera. Instead, we adopted a chest-mounted design with four cameras (Figure 3), each with a diagonal FOV of 160°. The mounted cameras provided an effective horizontal FOV of approximately 120°. The four cameras covered the forward, downward, left, and right views. The forward-facing camera monitored the direction of travel. The downward-facing camera covered the near-field ground, including curbs and steps. The left and right cameras captured lateral vehicle and pedestrian movement that is difficult to observe reliably from the forward view alone.

The device was connected to a standard smartphone through a USB cable. The smartphone acquired images from the four cameras independently through the USB UVC protocol and could enable each camera separately. An application on the smartphone communicated with a back-end computer through a server. This allowed the RSA to control which cameras were enabled in real time, view the corresponding images, and communicate with the VIP user through live audio. This study focused on how camera configuration affects the capabilities of ETAs. Therefore, we did not impose specific restrictions on the RSA’s role. Based on our previous work [18], the RSA could describe the scene ahead, provide short-distance walking guidance, or use a controller to send predefined instructions delivered as synthesized speech for immediate actions, such as stopping or veering slightly to the left.

We recruited four VIPs with varying degrees of visual impairment to use the prototype. In real street scenes in Zhuzhou, Hunan Province, China, participants walked along a predefined route with assistance from the RSA. In addition to walking on sidewalks, they also crossed busy intersections. During the demonstration, volunteers recorded video and provided safety support. When VIP users were walking with normal RSA assistance, the volunteers had no verbal communication or physical contact with the participants. The participants communicated only with the RSA.

This field demonstration was illustrative rather than evaluative and did not compare alternative camera configurations.

## 3. Results

### 3.1. Distribution of Urban Mobility Hazards

**Figure 4:**
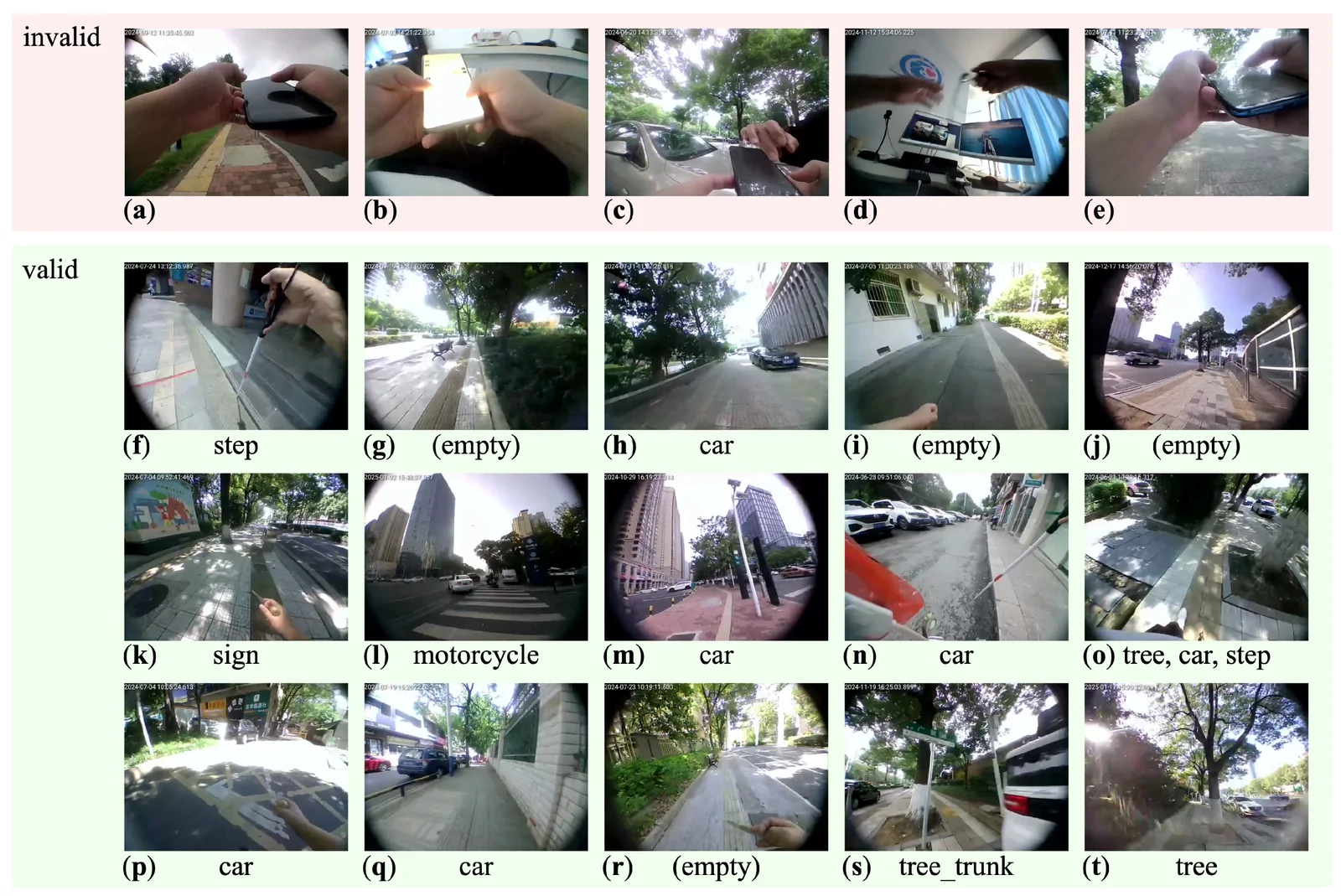
Examples of VLM output validity and hazard-category recognition. (**a**–**e**) The upper red panel shows invalid frames, including indoor scenes, hand-occluded frames, non-travel scenes, and frames with severe occlusion. (**f**–**t**) The lower green panel shows valid street-scene frames, with the text indicating the phrase lists produced by the VLM.

We collected 1486 min of video and sampled 114,736 images. Among the JSON outputs produced by the VLM, 886 samples contained invalid JSON text due to hallucination and were discarded. Another 32,497 samples were classified as invalid because they were not outdoor scenes or had insufficient image quality, leaving 81,353 valid samples. Manual inspection of sampled outputs showed that VLM-based hazard analysis had low recall and missed many hazards (Figure 4). Therefore, the hazard list used for simulation still had to be constructed manually. However, when spatial-relationship understanding was not required, the VLM identified the types of visible hazards with high accuracy. It was therefore suitable as a basis for multiple-frame sampling and helped the hazard list cover a broader range of hazard categories.

**Figure 5:**
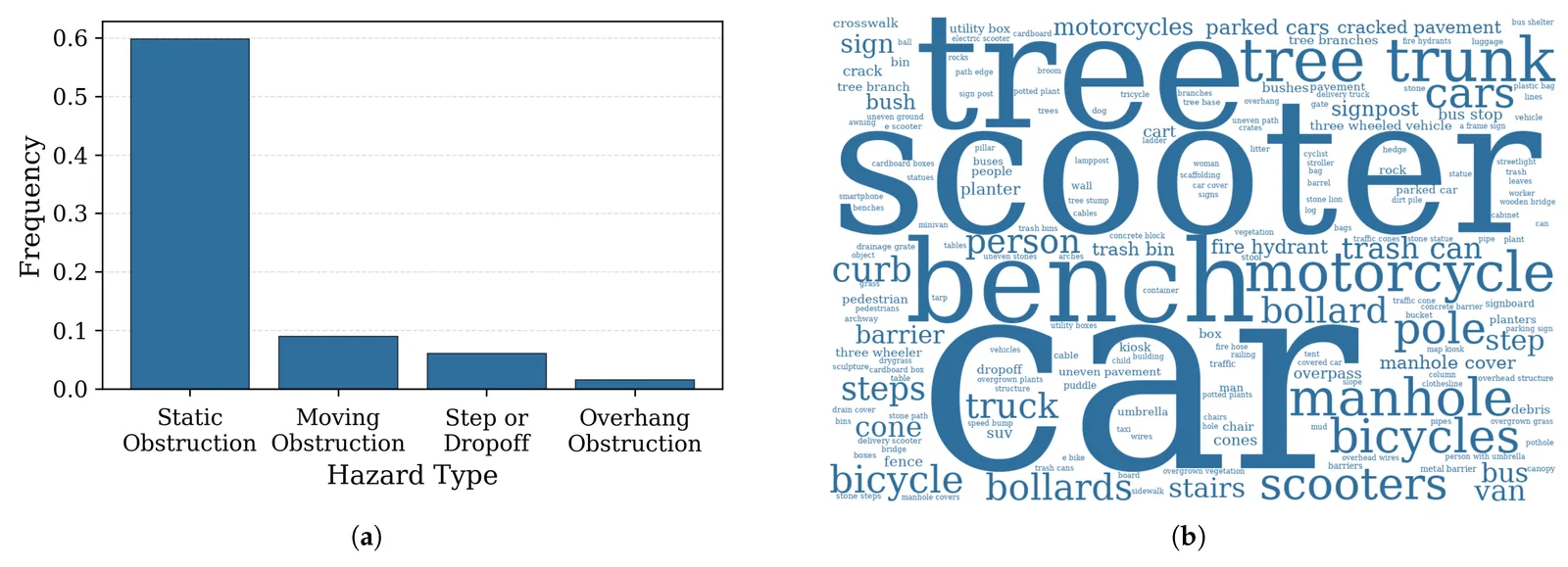
Distribution of urban mobility hazards identified with VLM assistance. (**a**) Occurrence frequencies of the four hazard categories among valid samples. Static obstructions were the most frequent, followed by moving obstructions, step or drop-off hazards, and overhanging obstructions. (**b**) Word cloud of raw hazard phrases produced by the VLM. High-frequency terms such as tree, car, scooter, bench, curb, and bollard reflect the composition of common hazard elements in real street scenes.

Among the valid samples, the occurrence frequency of each hazard category is shown in Figure 5a. To verify that the manually constructed hazard list aligned with the real environment, we generated a word cloud from the VLM outputs, as shown in Figure 5b. The word cloud shows that vehicle-related objects, represented by car and scooter, were the main hazards in urban travel environments. Sidewalk infrastructure, including tree, bench, and pole, represented common static obstruction sources. These object categories matched the hazard list obtained through manual sampling, as shown in Table 1. However, because manual annotation can be biased and automatic VLM labeling had low recall, the frequencies of some objects, such as curb and bench, differed from those in the word cloud. This discrepancy did not affect the subsequent selection of hazard categories for simulation because the simulation environment used only the specific hazard categories in the hazard list.

### 3.2. Visibility Trade-Offs Across Camera Configurations

**Figure 6:**
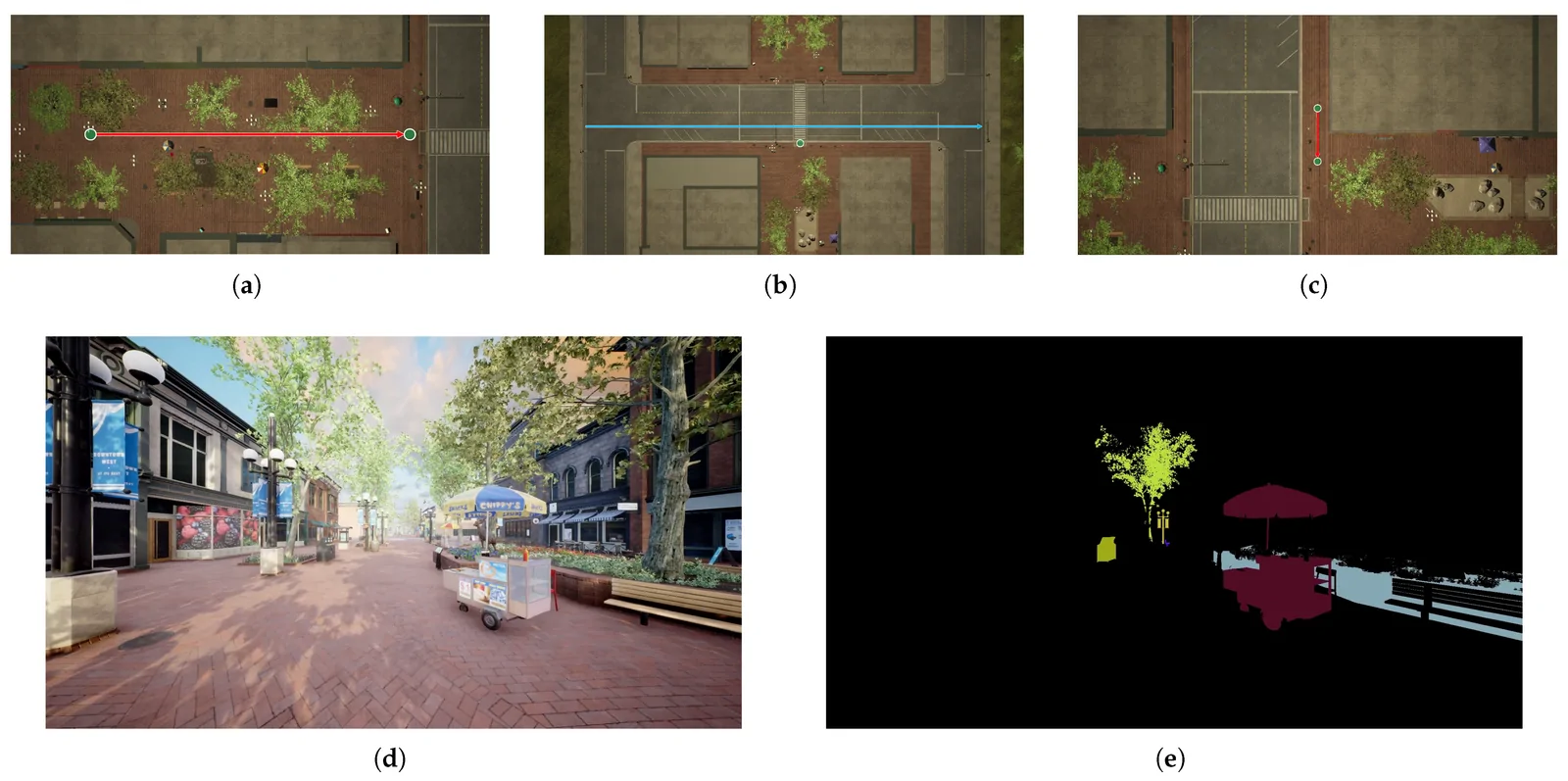
Urban simulation environments and semantic-segmentation output used to evaluate visibility under different camera configurations. (**a**) The first experiment used a walking route to evaluate FOV trade-offs for a forward-facing camera. (**b**) The second experiment used a vehicle approaching from the side to compare forward-facing and lateral camera coverage. (**c**) The third experiment used a route containing low near-ground hazards to compare forward-facing and downward-facing camera coverage. Red arrows indicate the direction of travel, and green dots indicate the start and end locations. (**d**) Example RGB street-scene image captured by the simulated camera. (**e**) Semantic-segmentation image from the same time point. Different colors represent different classes of environmental objects. This output was used to quantify the pixel coverage of hazard elements.

We built the urban simulation environment in Unreal Engine using the Downtown West Modular Pack. The layouts of the three experiments are shown in Figure 6a–c: the walking route used to evaluate forward-view FOV trade-offs, the lateral-vehicle scenario, and the route containing low near-ground hazards, respectively.

We used the output of the semantic-segmentation camera for analysis. For one representative instance from each typical hazard category in Table 1, we counted the number of pixels occupied by that instance in each semantic-segmentation frame. For multi-camera configurations, the system-level pixel count at each time point was defined as the maximum target pixel count across the synchronized camera views. The pixel count was then expressed as a function of time, producing a pixel-count–time profile. This profile characterizes both the visibility of a target during continuous motion and its temporal variation. It therefore provides a basic metric for comparing the hazard-sensing capability of different camera configurations. As an example, Figure 6e shows the semantic-segmentation output corresponding to the RGB image in Figure 6d.

Changes in pixel count over time reflect not only whether a target enters the camera view, but also how its image scale changes during continuous motion and how long it remains observable. Therefore, our data analysis focused on two aspects. First, we analyzed the pixel-count–time curve of each target to evaluate how its visibility changed under different camera configurations and when it reached an effective imaging scale. Second, we measured how long the target remained at or above a reference pixel-count threshold and recorded the first and last times at which it reached that threshold. These temporal measures describe the observation opportunity provided by each configuration.

**Figure 7:**
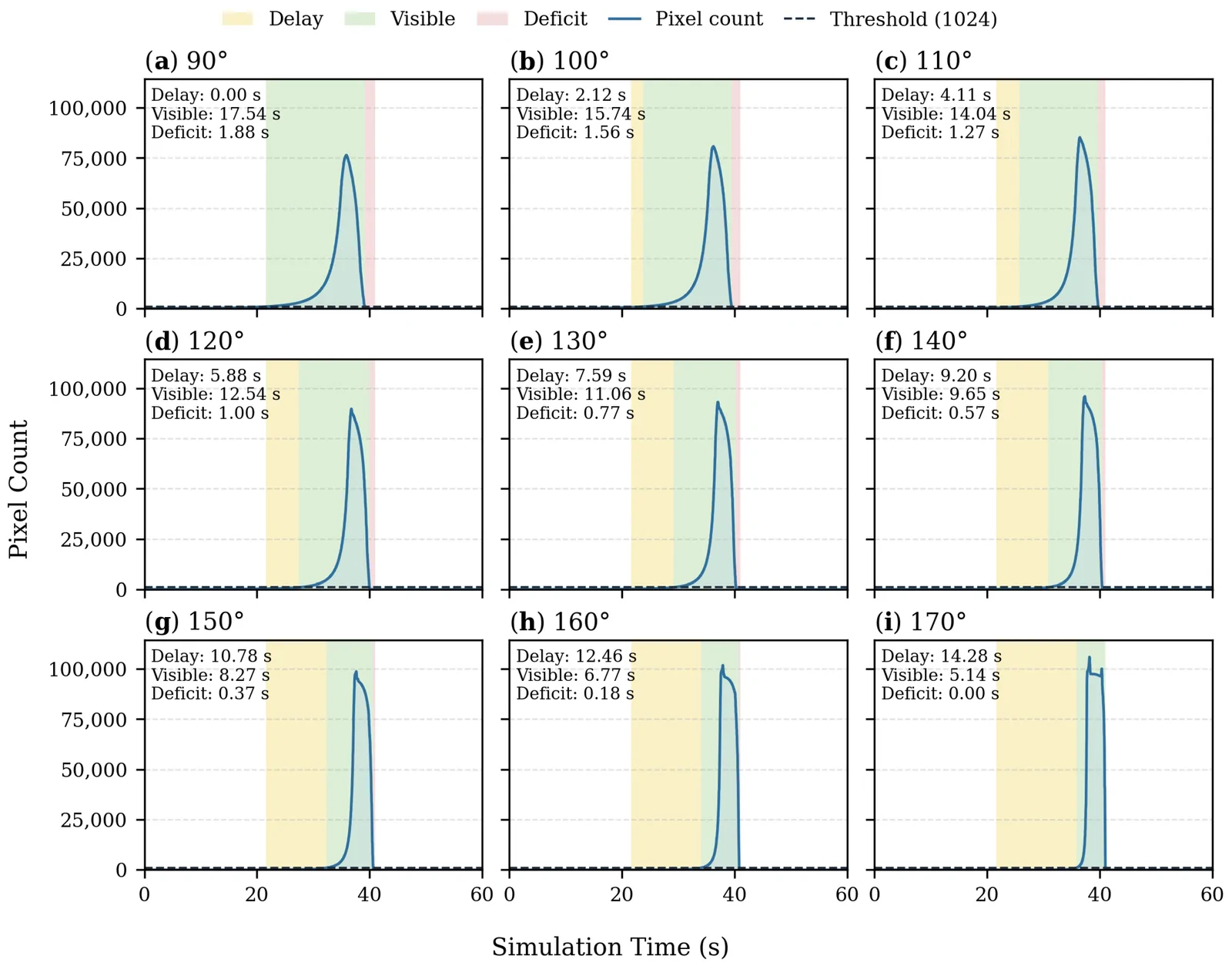
Target pixel count over time under different horizontal FOVs, using a bench as an example. (**a**–**i**) Horizontal camera FOVs from 90° to 170°. The blue curve indicates the semantic-segmentation pixel count of the target, and the dashed line indicates the 1024-pixel threshold. The yellow, green, and red shaded regions indicate the delay before reaching the threshold, the effective visible interval, and the observation gap caused by early disappearance, respectively.

Figure 7 shows the pixel-count–time curves for a bench, with 1024 pixels used as the threshold for marking the time interval during which the target met the 32×32-pixel requirement for reliable object recognition described in the Methods. The results show that, once the target entered the camera view, a smaller FOV usually reached the given pixel-count threshold earlier. This is because, at fixed resolution, a smaller FOV provides a higher angular sampling rate, allowing the target to obtain sufficient pixel coverage sooner. However, when the FOV was too small, the target also tended to leave the view too early. Its observable duration was therefore shorter than the actual time over which the camera passed the target. Only after the FOV increased to a certain range did the time at which the target disappeared become closer to the actual passing time, which better supported continuous and stable observation.

In addition, objects with different geometric forms and spatial extents showed clear differences in the time at which they left the camera view (see Supplementary Materials). Compared with compact targets, targets with greater lateral extent or more complex contours often showed larger changes in disappearance time across camera configurations. If a target left the view earlier than its reference baseline, the corresponding camera configuration provided insufficient sustained observation for that target. In this experiment, both the simulated route and the viewing direction were set under relatively ideal conditions, which should in principle provide sufficient observation opportunities. Therefore, if a target still left the view early under these conditions, it likely had a higher risk of being missed in real environments.

**Figure 8:**
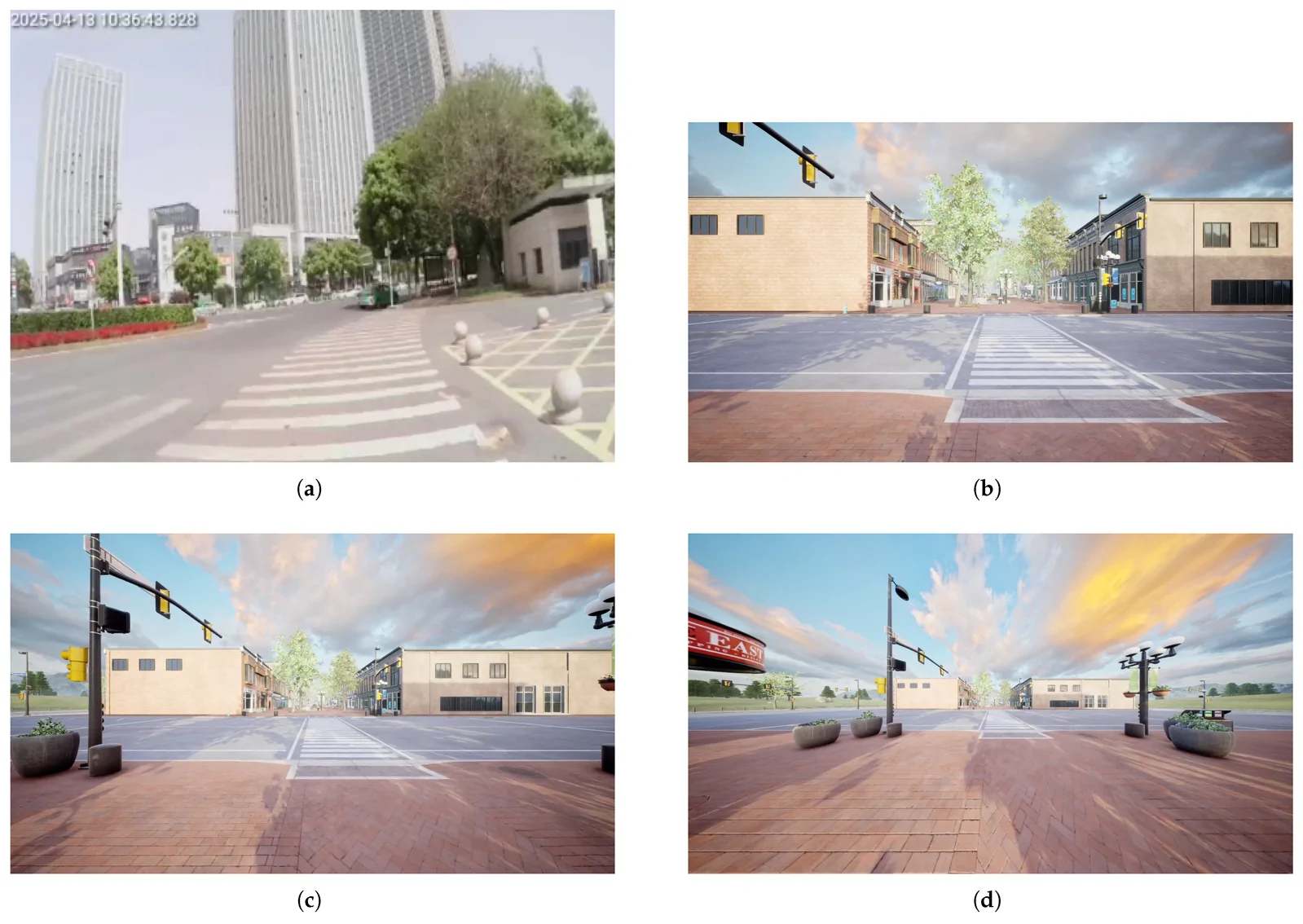
Comparison of a representative real-camera image with simulated images at different horizontal FOVs. (**a**) Forward-facing image captured by the prototype during the road-crossing demonstration, using a camera with a nominal diagonal FOV of 160° and an effective horizontal FOV of approximately 120°. (**b**–**d**) Simulated images captured at the same camera position with horizontal FOVs of 90°, 120°, and 150°, respectively. The simulated images followed an ideal pinhole projection.

To clarify the relationship between the prototype and simulated views and examine the image stretching associated with an ultra-wide horizontal FOV, Figure 8 compares a representative frame from the forward-facing camera of the prototype during the road-crossing demonstration with simulated images captured at horizontal FOVs of 90°, 120°, and 150°.

The prototype camera provided an effective horizontal FOV of approximately 120°, but the resulting image was not geometrically equivalent to the simulated image generated with a horizontal FOV of 120°. The difference in image stretching arose from the fisheye projection of the real camera and the ideal pinhole projection used in the simulation. Consequently, the real image may be closer in appearance to a pinhole simulation generated with a different horizontal FOV than to the 120° pinhole image.

**Figure 9:**
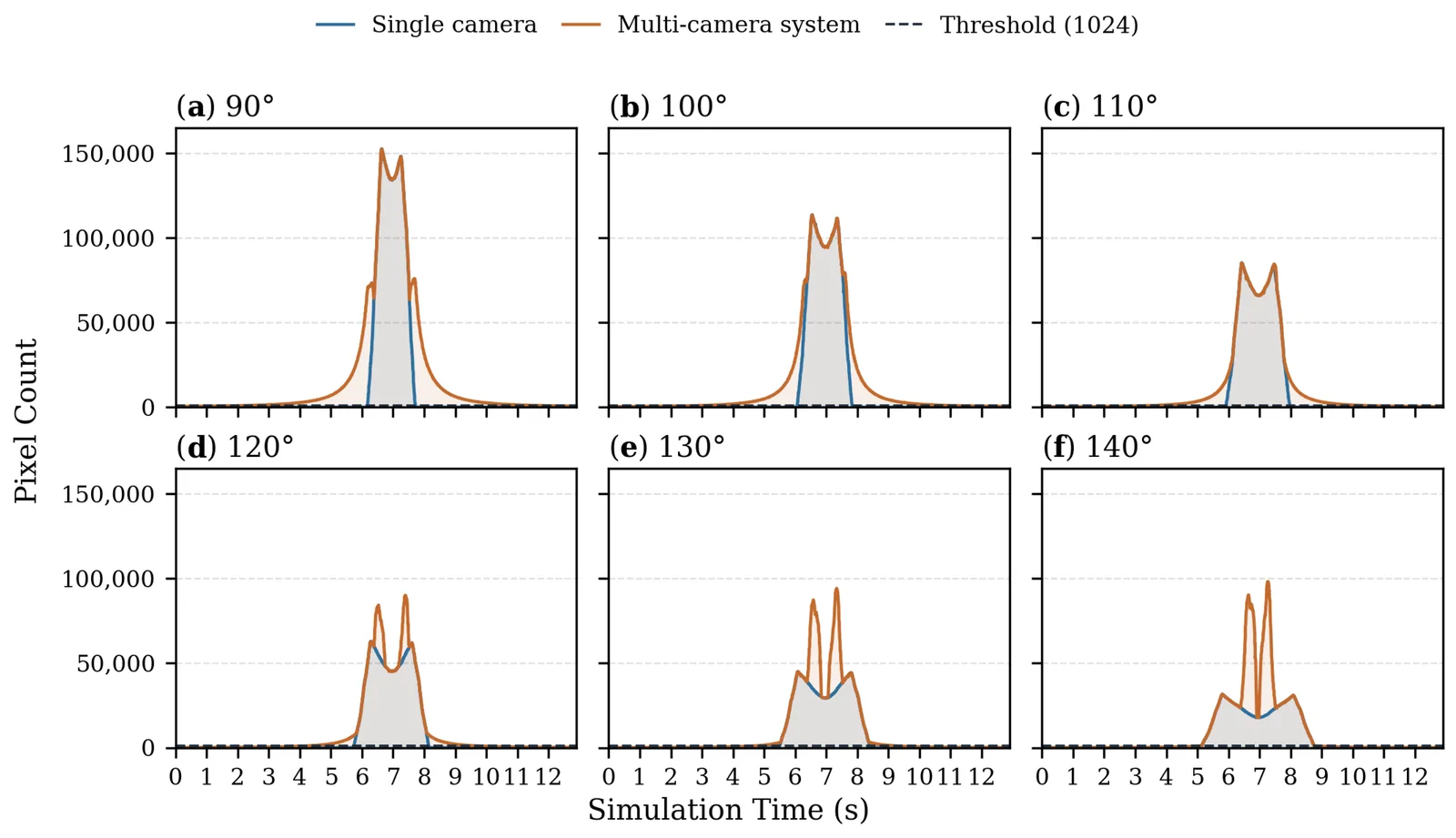
Target pixel count over time for a vehicle approaching from the side under the single forward-facing camera and the three-camera configuration. (**a**–**f**) Horizontal FOVs from 90° to 140° in 10° increments. The blue curve indicates the single-camera configuration, and the orange curve indicates the multi-camera configuration. The dashed line indicates the 1024-pixel reference threshold.

Because the first experiment showed pronounced perspective stretching at excessively wide FOVs (Figure 8d), the second and third experiments were restricted to horizontal FOVs from 90° to 140°. Figure 9 compares the single forward-facing camera with the three-camera configuration for the simulated vehicle approaching from the side. At a horizontal FOV of 120° and the 1024-pixel reference threshold, the vehicle reached the reference imaging scale 1.536 s earlier with the lateral views than with the forward-facing camera alone. This earlier observation was maintained across FOVs from 90° to 140°, although the gain decreased as the FOV increased.

**Figure 10:**
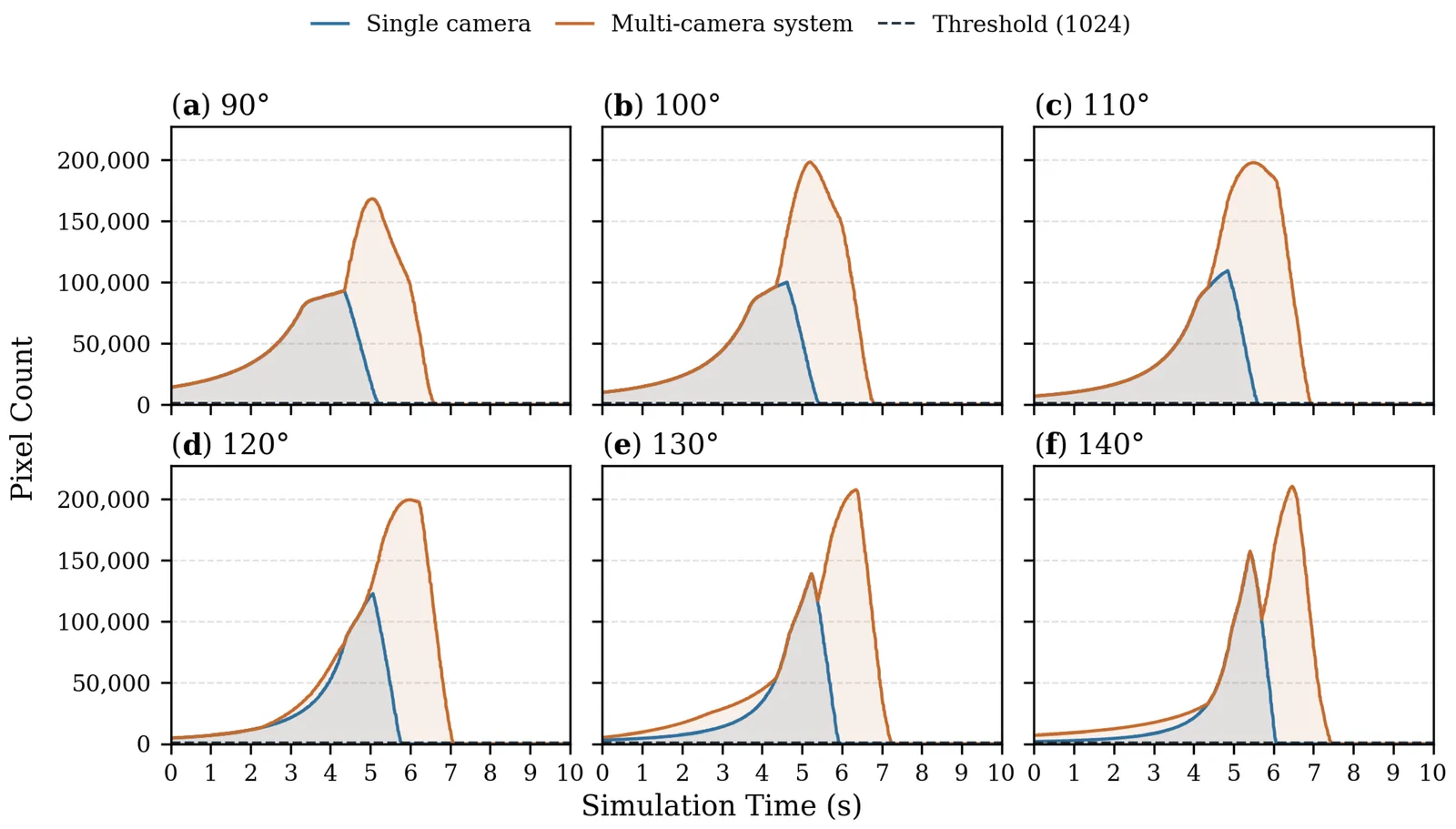
Target pixel count over time for a low bollard under the single forward-facing camera and the two-camera configuration covering the forward and downward views. (**a**–**f**) Horizontal FOVs from 90° to 140° in 10° increments. The blue curve indicates the single-camera configuration, and the orange curve indicates the multi-camera configuration. The dashed line indicates the 1024-pixel reference threshold.

Figure 10 compares the single forward-facing camera with the two-camera configuration for the low bollard. At a horizontal FOV of 120° and the 1024-pixel reference threshold, adding the downward view increased the effective visible duration from 5.775 s to 7.080 s and delayed the last threshold crossing by 1.302 s. The additional observation time varied little across the tested FOVs because changing the horizontal FOV of the downward-facing camera had little geometric effect on when effective observation of the low bollard ended.

### 3.3. Road-Crossing Demonstration of the Four-Camera Prototype

**Figure 11:**
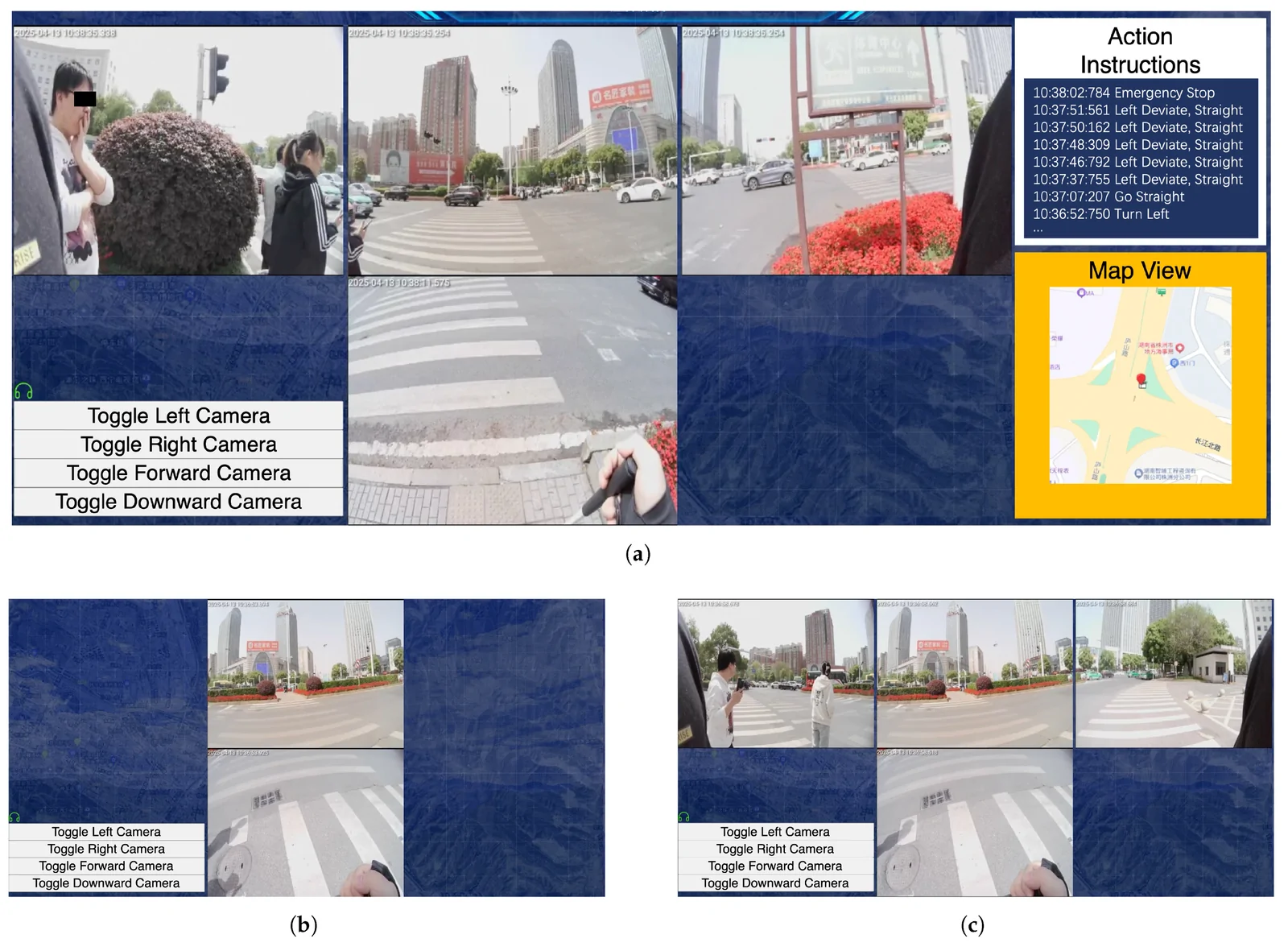
Multi-camera operating interface and view comparison used by the RSA during the field road-crossing demonstration. Images in this figure were manually edited to translate the user interface and protect the privacy of passersby. (**a**) The RSA interface displays multiple real-time video streams, camera toggles, a command log, and a map view. Chinese labels in the map view denote geographic place names provided by the map service and are not study-specific annotations. (**b**) When only the forward-facing and downward-facing cameras are enabled, the interface mainly provides information about the road ahead and the near-field ground. (**c**) After the left and right cameras are enabled, the RSA can simultaneously observe vehicles approaching from either side, pedestrians, and the intersection environment, providing a more complete basis for road-crossing decisions.

As shown in Figure 11a, the real-world street demonstration showed that, with RSA support, the four-camera prototype could provide information about the forward view, the near-field ground, and traffic from both directions for road-crossing decisions.

When participants crossed a busy intersection, the forward and downward views (Figure 11b) helped the RSA confirm the participant’s current position, initial walking direction, and the presence of steps, curbs, or other near-field ground hazards. However, these two views alone were insufficient for judging whether vehicles approaching from either side had fully passed. This limitation was especially clear when vehicles approached from either side, waited at the intersection, or had just moved out of the edge of the forward view. Forward-view information alone could not support a robust decision about when to start crossing. After the left and right cameras were enabled (Figure 11c), the RSA could observe vehicles from both directions more continuously as they approached, slowed down, waited, and passed. The RSA could also use nearby pedestrian behavior as additional evidence for deciding whether the participant should start crossing.

It is important to note that, even if traffic signals can be recognized accurately through improved hardware and algorithms, the decision to start crossing cannot be based on the signal alone. Vehicles may still be passing through the intersection, and some vehicles may violate the signal. Without the left and right cameras, the system has no direct way to perceive whether vehicles are approaching from either side and cannot make a reliable safety decision. With the left and right cameras, the RSA can more fully assess traffic from both directions, check whether any vehicles have not yet passed, and use the behavior of other pedestrians as additional decision support.

## 4. Discussion

The statistical analysis of real street-scene images showed a clear frequency distribution of mobility hazards faced by VIPs in urban environments. The word cloud in Figure 5b shows that, among the 81,353 valid samples obtained from 1486 min of video, hazard sources were concentrated in several high-frequency categories while also forming a rich long tail. Vehicles, scooters, and pedestrians were the main dynamic hazards in urban travel, whereas curbs, bollards, poles, and trees were the main static hazards. Step or drop-off hazards were also present. The hazard list obtained through multiple-frame sampling (Table 1) was consistent with high-frequency terms in the word cloud, including tree, car, scooter, bench, curb, and bollard. It also matched the static hazard types contained in the unmodified urban game assets. These findings indicate that the statistical analysis of street-scene images captured common hazard patterns in urban environments and that the selected simulation environment was representative of real-world conditions.

Ensuring safe mobility for VIPs remains challenging in complex urban traffic environments. ETAs can guide users away from hazards only if those hazards first fall within their sensing coverage. Varshney et al. [24] examined how sensor placement affects outdoor navigation algorithms. This study addresses an earlier design step by first summarizing hazards from real urban street scenes and then testing their visibility in simulation. We argue that sensing coverage is a necessary condition that must be satisfied before the performance of systems or algorithms can be meaningfully discussed. This is essential for designing ETAs that are practically usable for VIPs.

The simulation results showed that, for a forward-facing camera, FOV creates a trade-off between when a target enters the camera view and when it leaves. At fixed resolution, a smaller FOV usually allows the target to reach the reference imaging scale earlier. However, it also causes the target to leave the camera view earlier and shortens the sustained observation time. For example, for the bench in Figure 7, when the threshold was set to 1024 pixels, the 90° FOV produced the longest visible duration at 17.54 s, but also produced an early-disappearance gap of 1.88 s. With the 170° FOV, the visible duration decreased to 5.14 s, and the curve was close to rectangular, indicating severe distortion (see Supplementary Materials). Within this simulated route, the 120° FOV provided a reasonable trade-off. Its visible duration was 12.54 s, the early-disappearance gap decreased to 1.00 s, and the pixel-growth curve retained an observable gradual-approach process.

Within the simulated settings, the results suggest avoiding both overly narrow and overly wide FOVs. When the FOV is too small, a target can leave the camera view before the camera passes it, reducing sustained observation. When the FOV is too large, the target reaches the reference imaging scale later and the gradual pixel-growth process is compressed. In addition, cameras with ultra-wide FOVs reduce the angular sampling rate, and edge distortion in real images can further weaken the interpretability of key regions. However, this conclusion does not preclude the use of fisheye cameras for localization when required by the algorithm. Our conclusion is instead based on the fundamental mobility need of obstacle avoidance.

Further analysis of the first experiment showed that increasing the horizontal FOV produced progressively more abrupt target-image growth and increasingly pronounced perspective stretching, particularly above 140°. The mean slope was calculated as the increase in target pixel count divided by the interval from the first crossing of the 1024-pixel reference threshold to the pixel-count peak. At horizontal FOVs from 90° to 170° in 10° increments, the corresponding mean slopes, expressed in $10^{3}$ pixels/s, were 5.28, 6.37, 7.82, 9.54, 11.72, 14.43, 18.57, 26.09, and 45.36, respectively. For an ideal pinhole camera, horizontal projection follows $x=f_{x}\tan\phi$, and the local image scale at the edge relative to the center is therefore $\sec^{2}\left(\theta /2\right)$. Across the same FOV sequence, the corresponding edge-to-center scale ratios were 2.00, 2.42, 3.04, 4.00, 5.60, 8.55, 14.93, 33.16, and 131.65, respectively. Both measures increased across the full FOV range, with a pronounced increase above 140°. This increase is consistent with the perspective stretching visible at 150° in Figure 8d.

The second and third experiments evaluated how coverage in different viewing directions affected hazard visibility under controlled conditions. The second experiment showed that the vehicle reached the reference imaging scale earlier with the lateral views than with the forward-facing camera alone across horizontal FOVs from 90° to 140°, although the gain decreased as the FOV increased. The third experiment showed that adding the downward view increased the effective visible duration of the low bollard by approximately 1.3 s across the tested FOV range.

Guided by the hazard distribution and simulation trade-offs described above, we built an ETA prototype with four cameras, each with a horizontal FOV of approximately 120°. Together, the cameras covered the forward view, the near-field ground, and the left and right views. For wearable ETAs in urban environments, this configuration represents a promising way to combine target scale with complementary directional coverage. The forward-facing camera monitored the direction of travel. The downward-facing camera covered curbs, steps, and near-field ground hazards. The left and right cameras supplemented the forward view by capturing lateral traffic that is difficult to observe reliably from the front. In the road-crossing demonstration shown in Figure 11, the RSA had difficulty judging whether vehicles from both directions had fully passed when only the forward and downward views were available. After the left and right cameras were enabled, the RSA could continuously observe vehicles as they approached, waited, and passed, and could use nearby pedestrian behavior to decide when to start crossing. Compared with studies that focus on indoor object search or assume a fixed camera input [22,23], our prototype emphasizes simultaneous visibility of the forward, downward, left, and right views in urban road-crossing scenarios.

The simulation and field results should be interpreted in light of several methodological limitations. The simulations used predefined trajectories, controlled motion, and an undistorted pinhole camera model, with no repeated sampling across environments or users. The reported differences therefore describe the tested conditions and do not support statistical inference beyond them. The field demonstration provides qualitative support for the feasibility of the proposed design by showing that the prototype could deliver forward, downward, left, and right views to an RSA during real-world walking and road crossing. Because the demonstration involved four participants and did not include a controlled comparison with alternative camera configurations under equivalent real-world conditions, the relative performance of the proposed design remains to be examined in future studies.

Future work can further apply methods currently used in autonomous driving to ETA research and incorporate design principles from other wearable systems. This study evaluated hazard visibility and included one real road-crossing demonstration, but it did not include perception, planning, and control algorithms in a closed-loop evaluation. Future work can extend the workflow established here, from real-world hazard statistics to simulation-based visibility analysis. Potentially dangerous scenarios, such as lateral vehicle approach, curb or drop-off hazards, and near-field obstacle occlusion, can be repeatedly constructed in simulation so that algorithm outputs can also be evaluated in a reproducible way. Camera-configuration design for wearable ETAs can also be informed by mature virtual reality (VR) headset designs. VR devices must cover a horizontal FOV greater than 180°, as well as vertical coverage in the forward and downward directions. They also require camera systems that are easy to calibrate and model, enabling stable tracking of controllers or hands. These future studies may further clarify how multi-camera systems can provide sufficient sensing coverage while remaining stable, calibratable, computationally tractable, and capable of supporting real mobility decisions for VIPs.

## 5. Conclusions

Ensuring safe mobility for VIPs in complex urban traffic environments remains a major challenge. VIPs not only walk on sidewalks, but also encounter situations such as climbing or descending steps, crossing pedestrian overpasses, crossing roads, and moving through mixed pedestrian and bicycle traffic. If wearable ETAs cannot observe traffic approaching from the left and right, they face serious safety limitations at busy intersections. Taken together, the three simulation experiments showed that hazard visibility depended on both horizontal FOV and coverage in different viewing directions. In the first experiment, a horizontal FOV of approximately 120° balanced the earlier attainment of the reference imaging scale at narrower FOVs against the longer retention of the target in view at wider FOVs. Compared with the forward-facing camera alone, the lateral and downward views evaluated in the second and third experiments allowed a vehicle approaching from the side to reach the reference imaging scale earlier and increased the effective visible duration of a low bollard, respectively. These findings support coordinated multi-camera coverage as a promising design choice for wearable ETAs in urban environments. The road-crossing demonstration illustrated the feasibility of using the chest-mounted four-camera prototype to provide forward, downward, left, and right views under remote sighted assistance.

We believe that, as in autonomous driving, free mobility for VIPs requires more than sufficient sensing capability and embodied intelligence in wearable devices. RSA intervention may still be needed in some situations, and infrastructure such as urban camera systems may help compensate for coverage gaps. A complete solution to VIP mobility also requires social-level considerations. For example, an illuminated white cane could make VIP users more noticeable when traveling at night and help prevent collisions caused by being overlooked. Building on the methodology used in this study, future work can connect perception, planning, control, human–computer interaction, infrastructure, and social information exchange within simulation environments to build a complete validation loop for VIP mobility solutions.

## Figures and Tables

**Table 1:** Counts and relative frequencies of urban mobility hazard elements manually extracted from 100 images selected by multiple-frame sampling.

Hazard Element	Frequency	Relative Frequency (%)	Hazard Element	Frequency	Relative Frequency (%)
curb	43	22.28	bicycle	3	1.55
car	25	12.95	ashtray	2	1.04
bollard	24	12.44	sign	2	1.04
tree	21	10.88	bus shelter	2	1.04
pole	17	8.81	stepping stones	1	0.52
scooter	15	7.77	bush	1	0.52
person	12	6.22	traffic cone	1	0.52
bin	6	3.11	guardrail	1	0.52
planter	4	2.07	ramp	1	0.52
step	4	2.07	barrier	1	0.52
bench	3	1.55	bike rack	1	0.52
tree pit	3	1.55	–	–	–

## Data Availability

The data presented in this study and the source code are available on request from the corresponding author due to privacy reasons.

## Supplementary Materials

The following supporting information can be downloaded at: https://doi.org/10.5281/zenodo.20269038 (accessed on 11 September 2026). It includes supplementary pixel-count–time plots for representative hazard objects and simulation videos with RGB and corresponding semantic-segmentation outputs under different horizontal FOV settings.
